# CT Pulmonary Angiography: Increasingly Diagnosing Less Severe Pulmonary Emboli

**DOI:** 10.1371/journal.pone.0065669

**Published:** 2013-06-12

**Authors:** Andrew J. Schissler, Anna Rozenshtein, Michal E. Kulon, Gregory D. N. Pearson, Robert A. Green, Peter D. Stetson, David J. Brenner, Belinda D'Souza, Wei-Yann Tsai, Neil W. Schluger, Andrew J. Einstein

**Affiliations:** 1 Department of Medicine, Columbia University Medical Center and NewYork-Presbyterian Hospital, New York, New York, United States of America; 2 Department of Radiology, Columbia University Medical Center and NewYork-Presbyterian Hospital, New York, New York, United States of America; 3 Department of Emergency Medicine, Columbia University Medical Center and NewYork-Presbyterian Hospital, New York, New York, United States of America; 4 Department of Biomedical Informatics and Department of Medicine, Columbia University Medical Center and NewYork-Presbyterian Hospital, New York, New York, United States of America; 5 Center for Radiological Research, Columbia University Medical Center and NewYork-Presbyterian Hospital, New York, New York, United States of America; 6 Department of Biostatistics, Mailman School of Public Health, Columbia University, New York, New York, United States of America; 7 Division of Pulmonary, Allergy, and Critical Care Medicine, Department of Medicine, Columbia University Medical Center and NewYork-Presbyterian Hospital, New York, New York, United States of America; 8 Cardiology Division, Department of Medicine, Columbia University Medical Center and NewYork-Presbyterian Hospital, New York, New York, United States of America; Sapienza University of Rome, Italy

## Abstract

**Background:**

It is unknown whether the observed increase in computed tomography pulmonary angiography (CTPA) utilization has resulted in increased detection of pulmonary emboli (PEs) with a less severe disease spectrum.

**Methods:**

Trends in utilization, diagnostic yield, and disease severity were evaluated for 4,048 consecutive initial CTPAs performed in adult patients in the emergency department of a large urban academic medical center between 1/1/2004 and 10/31/2009. Transthoracic echocardiography (TTE) findings and peak serum troponin levels were evaluated to assess for the presence of PE-associated right ventricular (RV) abnormalities (dysfunction or dilatation) and myocardial injury, respectively. Statistical analyses were performed using multivariate logistic regression.

**Results:**

268 CTPAs (6.6%) were positive for acute PE, and 3,780 (93.4%) demonstrated either no PE or chronic PE. There was a significant increase in the likelihood of undergoing CTPA per year during the study period (odds ratio [OR] 1.05, 95% confidence interval [CI] 1.04–1.07, P<0.01). There was no significant change in the likelihood of having a CTPA diagnostic of an acute PE per year (OR 1.03, 95% CI 0.95–1.11, P = 0.49). The likelihood of diagnosing a less severe PE on CTPA with no associated RV abnormalities or myocardial injury increased per year during the study period (OR 1.39, 95% CI 1.10–1.75, P = 0.01).

**Conclusions:**

CTPA utilization has risen with no corresponding change in diagnostic yield, resulting in an increase in PE detection. There is a concurrent rise in the likelihood of diagnosing a less clinically severe spectrum of PEs.

## Introduction

Acute pulmonary embolism (PE) is a common disease with potentially high morbidity and mortality [1]–[3]. Prompt diagnosis and treatment with anticoagulation has been shown to improve outcomes significantly [4]. Over the past decade, multidetector-row computed tomography pulmonary angiography (CTPA) has become the primary tool for the diagnosis of PE [5]. CTPA is non-invasive, takes minutes to perform, and has very high sensitivity and specificity [6]–[9]. It is therefore not surprising that CTPA utilization has grown rapidly. National data suggests a steady increase in the total number of computed tomography scans performed in the emergency department (ED) [10] and several CTPA-specific studies at individual hospital centers support these trends [11]–[15].

Increased CTPA utilization has led to a corresponding rise in PE detection [16]–[18]. This has engendered growing concern about over-diagnosis: CTPA may be detecting more PEs of minimal clinical significance [16]. Two epidemiological studies – one using national data and another using data from the state of New York – showed a rise in PE incidence since the introduction of CTPA with minimal change in mortality [16], [17]. A recent study using individual patient-based data demonstrated an increased incidence of PE with no significant change in 7 or 14 day overall mortality [18]. These studies suggest that we are increasingly detecting a different, less fatal form of disease with CTPA.

This study was designed to provide additional supporting evidence that increased CTPA utilization has resulted in an increased detection of a less severe disease spectrum. In addition to reviewing patterns of CTPA utilization and diagnostic yield (percent of scans positive for an acute PE), we assessed trends in PE disease severity, measured by the presence of right ventricular dysfunction or dilatation on echocardiography and myocardial injury on cardiac troponin testing. The absence of right ventricular abnormalities and myocardial damage in patients with an acute PE identifies a subset of patients with milder disease who could be considered candidates for outpatient management [19].

## Materials and Methods

This study was performed at NewYork-Presbyterian Hospital/Columbia University Medical Center. The study was approved by the Columbia University Medical Center Institutional Review Board with a waiver of informed consent.

### Data Collection and Processing

We retrospectively reviewed electronic medical records and identified all adults (age≥18 years old) who underwent CT angiography of the chest in our ED between 1/1/2004 and 10/31/2009. Data from 11/1/2009–12/31/2009 could not be included because of a change in our picture archiving and communication system (PACS). All CT angiograms were performed on the same scanner (Siemens Somatom Sensation 10, Siemens Medical Solutions USA, Inc., Malvern, PA) during the study period. We collected data on patient age, sex, race/ethnicity, troponin I levels, transthoracic echocardiogram (TTE) findings, CT angiogram findings, emergency room disposition (e.g. discharged, admitted), and mortality during hospital stay if admitted. We also collected data on total ED visits for each year in the study period; daily data was obtained for 2009. The data was compiled in a MySQL database (version 5.1.43; Oracle Corporation, Redwood Shores, CA).

A total of 5,088 CT angiograms were initially identified. All CT angiograms ordered for an indication other than to assess for PE, such as aortic dissection, were excluded (550 studies). A total of 323 patients underwent more than one CTPA during our study period. For these patients, we limited our analysis to only the first CTPA (428 studies excluded) given concern that a few patients with multiple scans in a short period of time could bias our results. Final radiologist reports could not be retrieved for 32 studies, and these were re-read by an attending chest radiologist (A.R.). If the images were not available, the study was excluded (5 studies). We excluded technically limited studies where the presence or absence of an acute PE was unclear to the attending radiologist dictating the final report (57 studies). Thus, a total of 4,048 CTPAs were included in the primary analysis.

To facilitate analysis in the context of the large percentage of negative scans, we developed a natural language processing algorithm in PHP: Hypertext Preprocessor (release 5.2, The PHP Group, http://www.php.net) to categorize all CTPA final reports as negative, positive, or unclear. All reports classified by the algorithm as “unclear” and “positive” were manually reviewed by one of the investigators (A.J.S.) and if there was any uncertainty by an attending chest radiologist (A.R.). Examinations that demonstrated evidence of an acute PE were categorized as positive. Studies were categorized as chronic PE if described as such by the attending radiologist in the final report (e.g. based on clot appearance within the vessel, such as a peripheral, crescent-shaped filling defect) [20]. Studies that showed no clear evidence of acute PE were categorized as negative. The automated classification of negative scans was validated by a senior radiology resident (M.K.) and an attending chest radiologist (A.R.) who reviewed reports from 100 randomly selected studies classified as negative, yielding 0 errors.

We manually reviewed all TTE final reports that resulted within 3 days of a positive CTPA scan. We specifically assessed right ventricular (RV) size and systolic function. Reports that concluded normal or “borderline” RV size and normal or “borderline” RV function were categorized as negative. Reports that concluded at least “mildly” dilated RV or at least “mildly” reduced RV systolic function were categorized as positive. Reports that were technically limited with no comment on RV size or function in the official report were categorized as inconclusive.

We reviewed myocardial-specific troponin levels for all CTPA scans positive for an acute PE. The peak troponin level that resulted within 24 hours of a positive CTPA was included in the analysis. Our hospital used 3 different assays during the study period. We set the cutoff for a positive troponin according to the manufacturer-determined cutoff for an acute myocardial infarction for each of the assays. Here, by “myocardial injury” we refer specifically to myocardial injury as indicated by a positive troponin test [21]–[24].

### Statistical Analysis

All data was exported from MySQL for statistical analysis in Stata/SE 11.0 (StataCorp, College Station, Texas). Descriptive statistics and chi-square tests were performed to compare annual population demographic data. We performed multivariate logistic regression analyses controlling for patient demographics (age, sex, race/ethnicity) to assess for any trends in CTPA utilization, diagnostic yield, and PE disease severity.

## Results

### CTPA Utilization Patterns and Diagnostic Yield

Of the 4,048 CTPAs from January 1, 2004 through October 31, 2009 included in the analysis, 268 were positive for an acute PE (6.62%), 30 were positive for a chronic PE (0.74%), and 3,750 were negative for an acute or chronic PE (92.64%).

The annualized study cohort demographic data is summarized in Table 1. There was no significant change in the study population with regard to sex and age during the study period. Race/ethnicity distributions changed significantly (P<0.01) during the study period, notably in 2009 when there was an increase in Other and corresponding decrease in Black, Hispanic and White.

**Table 1:** Demographic data for subjects who underwent a CTPA in the ED.

Characteristic	Study Year						
	2004	2005	2006	2007	2008	2009*	P-value
Total CTPAs Performed	506	626	717	736	778	685	
Age (years)							0.57
Mean	53.3	53.8	55.1	54.9	54.4	54.4	
SD	18.8	19.5	19.2	19.3	19.6	19.2	
Race/Ethnicity (%)							<0.01
White	18.4	17.4	16.1	18.0	14.7	15.7	
Black	21.8	24.1	21.4	22.5	21.8	16.9	
Hispanic	29.8	31.1	32.3	30.8	28.8	21.3	
Other	30.0	27.5	30.2	29.2	34.7	46.2	
Sex (%)							0.90
Male	32.8	34.5	32.8	35.1	32.7	33.3	
Female	67.2	65.5	67.2	65.0	67.4	66.7	

* Incomplete year: includes data from Jan. 1, 2009 to Oct. 31, 2009.

**Figure 1:**
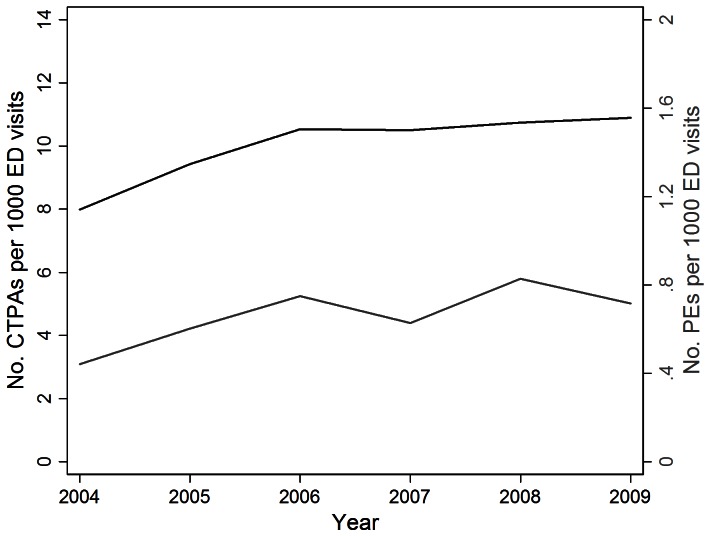
Annual CTPA utilization patterns. Graph shows an increase in the number of CTPAs ordered from 2004 to 2009 with concurrent increase in number of scans positive for an acute PE.

Figure 1 shows annual CTPA utilization, defined as number of studies per 1000 ED visits, increased significantly between 2004 and 2009 (odds ratio [OR] 1.05, 95% confidence interval [CI] 1.04–1.07, P<0.01). In 2004 there were 8.0 CTPAs performed per 1000 ED visits and this increased to 10.9 in 2009.

Diagnostic yield, or percent of all analyzed CTPAs positive for an acute PE, averaged 6.5% (range 5.5% to 7.7%, standard deviation [SD] = 0.79) with no significant change during the study period (OR 1.03, 95% CI 0.95–1.11, P = 0.49). Age was associated with an increased likelihood of a positive CTPA (OR 1.01, 95% CI 1.01–1.02, P<0.01). Females were less likely than males to have a positive CTPA (OR 0.76, 95% CI 0.59–0.99, P = 0.04). There was no statistically significant relationship between race/ethnicity and likelihood of a positive study. Table 2 contains annualized utilization and diagnostic yield data. Table 3 summarizes the association of demographic variables with diagnostic yield.

**Table 2:** Annualized CTPA utilization, diagnostic yield and disease severity data.

	Study Year					
	2004	2005	2006	2007	2008	2009*
Total ED visits	63357	66397	68076	70070	72430	62854
Total CTPAs performed	506	626	717	736	778	685
Positive CTPAs	28	40	51	44	60	45
Diagnostic yield (% total CTPA positive)	5.5	6.4	7.1	6.0	7.7	6.6
Total CTPAs with troponin level analyzed	25	36	39	35	49	38
CTPAs with negative troponin	18	31	36	28	44	32
% CTPAs with troponin levels analyzed with negative troponin	72.0	86.1	92.3	80.0	89.8	84.2
Total CTPAs with TTE analyzed	20	27	33	31	40	33
CTPAs with TTE demonstrating no RV abnormalities (negative)	5	8	18	12	18	15
% CTPAs with TTE analyzed with negative TTE	25.0	29.6	54.5	38.7	45.0	45.5
Total CTPAs with troponin level and TTE analyzed	18	26	27	27	36	28
CTPAs with negative troponin and negative TTE	3	7	13	10	16	13
% CTPAs with TTE & troponin level analyzed with both negative	16.7	26.9	48.1	37.0	44.4	46.4

* Incomplete year: includes data from Jan. 1, 2009 to Oct. 31, 2009.

**Table 3:** Association of various demographic variables and likelihood of a positive CTPA scan.

		OR	95% CI		P-value
Year		1.03	(0.95	1.11)	0.49
Age		1.01	(1.01	1.02)	<0.01
Female		0.76	(0.59	0.99)	0.04
Race	*Black*	1	–	–	–
	*Hispanic*	0.75	(0.52	1.10)	0.14
	*Other*	1.03	(0.73	1.45)	0.88
	*White*	0.98	(0.66	1.46)	0.93

### PE Disease Severity

Of the 268 CTPAs positive for an acute PE, 184 had an associated TTE (68.6%) and 222 had an associated troponin level (82.8%). 162 patients (60.5%) had both a TTE and troponin level, while 24 patients (9.0%) had neither. The likelihood of having a TTE did not change during the study period (OR 1.03, 95% CI 0.87–1.22, P = 0.70). The likelihood of having a troponin level did not change during the study period (OR 0.91, 95% CI 0.73–1.14, P = 0.42). The likelihood of having both a TTE and troponin level did not change during the study period (OR 0.98, 95% CI 0.83–1.15, P = 0.78).

**Figure 2:**
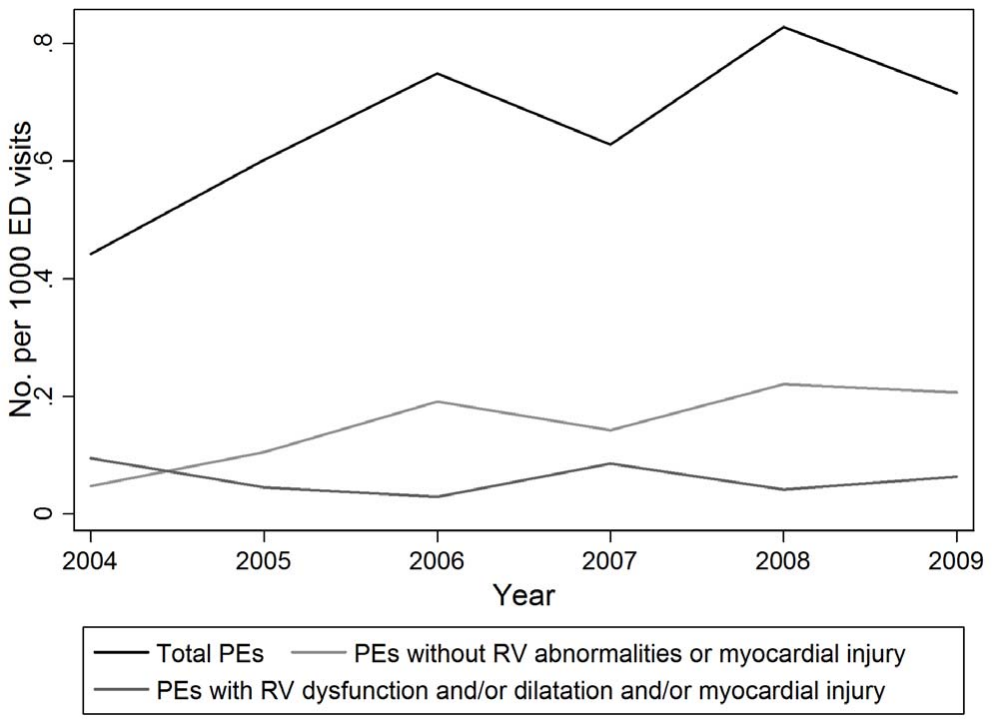
Annual number of PEs diagnosed by CTPA, number of PEs without associated RV abnormalities or myocardial injury, and number of PEs with associated RV dysfunction and/or dilatation and/or myocardial injury.

Figure 2 shows trends in PE disease severity during the study period. In patients diagnosed with an acute PE on CTPA, there was a significant increase in the likelihood of having less severe disease (i.e. no RV dysfunction or dilatation or myocardial injury) per year during the study period, adjusted for age, race/ethnicity, and sex (OR 1.39, 95% CI 1.10–1.75, P = 0.01). There was a downward trend in the likelihood of having either RV dysfunction or dilatation or troponin level elevation during the study period, adjusted for age, race/ethnicity, and sex, which approached statistical significance (OR 0.81, 95% CI 0.66–1.01, P = 0.06). There were no statistically significant relationships noted between disease severity and age, sex, or race. Table 2 contains annualized disease severity data. Table 4 summarizes the association of demographic variables with disease severity.

**Table 4:** Association of various demographic variables and likelihood of diagnosing a less severe disease spectrum.

		OR	95% CI		P-value
Year		1.39	(1.10	1.75)	0.01
Age		0.98	(0.97	1.00)	0.12
Female		0.71	(0.35	1.42)	0.33
Race	*Black*	1	–	–	–
	*Hispanic*	1.41	(0.51	3.85)	0.51
	*Other*	0.42	(0.16	1.10)	0.08
	*White*	0.71	(0.25	2.00)	0.52

All patients in our study diagnosed with an acute PE were admitted for further management. A total of 20 patients (7.5%) out of 268 diagnosed with an acute PE on CTPA died prior to discharge. In-hospital mortality was 3.2% among patients with no RV abnormalities or myocardial injury compared to a mortality rate of 14.1% among patients with RV dysfunction and/or dilatation and/or myocardial injury.

Sensitivity analyses excluding 2009 given the significant change in racial demographics and incomplete dataset (November and December were excluded) showed no significant change in our results.

## Discussion

CTPA utilization in the ED rose from 2004 to 2009 without a significant change in diagnostic yield. As a result more PEs were diagnosed, which is in agreement with published data that reflects an upward trend in disease incidence [16]–[18]. Recent studies have suggested this rise may be attributed to the phenomenon of “over-diagnosis,” (the detection of minimally clinically significant disease including a certain number of false positives) [16]–[18]. Our study further supports this premise and demonstrates that increased CTPA utilization has resulted in the detection of more PEs with a less severe disease spectrum.

We used RV dysfunction or dilatation and myocardial injury as surrogates for disease severity. The presence of RV dysfunction or dilatation on TTE in normotensive patients signifies an increased risk of recurrent PE and death. Multiple studies report mortality rates between 4.3–12.8% in normotensive patients with acute PE and RV dysfunction or dilatation, compared to less than 1% in the absence of RV dysfunction or dilatation [19], [25], [26]. Unfortunately, the definition of RV dysfunction or dilatation differs in these studies and includes various classifications [19], [25], [26]. It is reasonable, though, to assume a completely normal study is indicative of a low-risk PE.

Myocardial damage, as evident by a critically elevated troponin I level in the setting of an acute PE, has been strongly associated with more severe disease. Two prospective studies of patients with acute PEs demonstrated a significant association between high troponin levels (levels indicative of myocardial injury) and overall mortality and complicated in-hospital course [27], [28]. A more recent retrospective study in hemodynamically stable patients similarly noted in-hospital mortality to be significantly increased in patients with high troponin levels compared to those with normal levels [29]. No significant difference in mortality was noted for patients with only moderately elevated troponin levels when compared to patients with normal troponin levels [29].

It should be noted that elevated troponin levels and RV abnormalities on TTE each provide independent negative prognostic significance [19], [25], [29]. The absence of RV dysfunction or dilatation on TTE combined with a normal or only moderately elevated troponin level identifies a patient group with a more favorable prognosis. This subset group has a significantly lower rate of in hospital mortality, as supported by our data, and complications [27], [29]. Our data strongly suggests that increased detection of this “low-risk PE” subgroup is significantly contributing to the overall rise in PE detection.

We did not include clot burden in our analysis as recent studies have suggested this is not predictive of short- or long-term mortality [30], [31]. The current thought is that the patient’s underlying cardiopulmonary reserve plays a major role in how well a given PE is tolerated. Moreover, PEs are associated with the release of pulmonary artery vasoconstrictors that account for at least some of the hemodynamic consequences [32].

Our data also demonstrates that older patients are more likely have a positive CTPA. This was expected as age is a known risk factor for the development of PE [33]. There is no statistically significant association between age and disease severity in our study. Interestingly, female patients are less likely to have a positive CTPA, but when the scan is positive for acute PE, females are just as likely as males to have less severe disease. There is no statistically significant relationship between race/ethnicity and diagnostic yield or disease severity.

There were a number of limitations to our study. The retrospective design limits the data available for analysis. For example, the hemodynamic status of our study population is unknown. Most patients were likely hemodynamically stable since they were able to undergo CTPA. We also do not have complete risk-factor profiles for our study population. This does not permit evaluation of the clinical appropriateness of the ordered CTPAs by computing each subject’s Wells score [34], [35] or Geneva score [36]. Our diagnostic yield is consistent with those published in similar studies suggesting that our ordering patterns are similar to those at other centers in the United States [11], [12], [14], [32]. We are further unable to assess any changes in the ED population over time that could have alternately increased the incidence of PE, for example an increase in oncology patients. Approximately 39% of subjects did not have both TTE and troponin level data for our final analysis. The rate of ordering of these studies was stable during the study period. We suspect that patients that did not undergo TTE or troponin testing were more likely to have had less severe disease. We set the cutoff for a positive troponin according to the level indicative of myocardial infarction because one of the assays did not permit a lower threshold. This is higher than the cutoff used in many PE studies [37], but the data suggests that higher troponin levels are more indicative of a worse prognosis even when compared to moderately elevated levels [27], [29]. There exists the possibility of some imprecision when directly comparing the cutoff points between the 3 troponin assays used in this study, but we suspect this to be limited. We did not include CTPA measurements of RVD as our radiologists did not consistently assess for this during the study period. There is data to suggest that quantitative CT parameters along with serum troponin measurements can be used as an alternative to transthoracic echocardiography (TTE) for the detection of RVD [38]. It is unclear why there was a significant change in our study population race/ethnicity demographic in 2009. This may be related to a change in how the data was either collected or recorded, but this could not be determined. Sensitivity analyses excluding 2009 data did not significantly change our results. We do not discuss utilization of other imaging modalities such as lung scintigraphy (V/Q scan) [39], magnetic resonance angiography [40], or conventional pulmonary angiography as our data are limited to PEs diagnosed by CTPA. Finally, our study conclusions may not be generalizable to all practice settings, which may vary in terms of spectrum of pathology and diagnostic study ordering patterns.

The increase in CTPA utilization necessitates a shift in thinking regarding the diagnosis and management of PE. As opposed to being a ruled-in or ruled-out diagnosis, it should rather be viewed as a disease spectrum. Some filling defects detected on CTPA are likely either false positive results, or clinically insignificant. Other filling defects carry high morbidity and mortality risk. Future studies should establish if the risks of therapeutic anticoagulation outweigh the benefits in smaller filling defects with no associated RV abnormalities or myocardial injury in the setting of no detectable deep venous thrombosis. Ultimately, it will be important to develop a grading system using objective clinical data (vital signs, laboratory data, and imaging results) with associated clinical pathways that guide decision making, including inpatient versus outpatient management as well as duration of anticoagulation treatment.
